# The Occurrence and Risk Assessment of Aflatoxin M_1_ in Cheeses Samples from Hamadan, Iran

**DOI:** 10.22037/ijpr.2020.112399.13754

**Published:** 2020

**Authors:** Amir Sasan Mozaffari Nejad, Ali Heshmati, Tayebe Ghiasvand

**Affiliations:** a *Department of Microbiology, Nutrition Health Research Center, Hamadan University of Medical Sciences and Health Services, Hamadan, Iran. *; b *Department of Nutrition and Food Safety, Nutrition Health Research Center, Hamadan University of Medical Sciences and Health Services, Hamadan, Iran.*

**Keywords:** Aflatoxin M_1_, Cheese, ELISA, Iran, Risk assessment

## Abstract

Aflatoxin M_1_ (AFM_1_) is a category of poisonous compounds found in milk and dairy products. The target of our research is to determine incidence and risk assessment AFM_1_ through the consumption of cheese in Hamadan province of Iran. Seventy cheese samples including cream cheese (n = 30) and Iranian white cheese (n = 40) were collected from different regions of Hamadan province, Iran and tested for AFM_1 _by ELISA technique. The estimated daily intake (EDI) and hazard index (HI) of AFM_1_ was determined. AFM_1 _was detected in 67 (95.7%) samples, including 39 (97.5%) Iranian white cheese (mean: 115.16 ng/kg; range: < 5-287 ng/kg) and 28 (93.3%) cream cheese samples (mean: 141.20 ng/kg; range: < 5-289 ng/kg). The level of AFM_1_ in 10% samples was above the maximum tolerance limit (250 ng/kg). EDI of AFM_1_ through cheese for a preschool child, an adult female, and an adult male was 0.138, 0.076, 0.065 ng/kg bw/day, respectively. In our study, HI for these groups was 0.690, 0.378 and 0.324, respectively. Although the incidence of AFM_1_ in cheese samples was high, the results, regarding risk assessment which indicated potential risks for the liver cancer among Iranian consumers due to the cheese consumption, are not concerned.

## Introduction

Mycotoxins are one of the primary natural chemical compositions that can be a serious concern all over the world, specially an international trade. Between 300 various mycotoxins, aflatoxins (AFs) are poisonous, cancerous, and primary classes of mycotoxins. They are fungal secondary metabolites which are frequently produced by some *Aspergillus* species, especially *A. nomius*, *A. flavus,* and *A. parasiticus*. They can be found in agricultural crops, milk (breast and animal), and dairy products under suitable conditions of humidity and temperature (1-7). 

Cheese is the major origin of aflatoxins among milk products for the fact that AFM_1_ is related to the casein fraction in milk which is nearly concentrated in cheese (3, 8). Researchers have demonstrated that the concentration of AFM_1_ is about three fold greater in various soft cheeses and around five fold greater in hard cheeses than in milk from which the cheese is manufactured (8).

Several studies from various countries have conducted about the occurrence of AFM_1_ in dairy products and suggested a permissible limit for it. These regulations differ between several countries with respect to economic considerations. Hence, the European Commission (EC) and the Institute of Standards and Industrial Research of Iran (ISIRI) have set a limit of 250 ng/kg for AFM_1_ in cheese variety (9-11). 

One of the most important methods for the estimation of liver cancer risk as result of AFM_1_ intake is to assess the risk of exposure to this mycotoxin (12, 13). For this purpose, its estimate daily intake (EDI) and hazard index (HI) were calculate and expressed. If HI value was lower than one, it means AFM_1_ intake from analysed products did had health risk for consumers (14).

ELISA and lateral flow strips are routine methods for AFM_1_ detection in many group numbers of samples; however, High-performance liquid chromatography (HPLC) with fluorescence detection (FD) is another confirmatory method for this purpose. In Iran, the ELISA technique is the most usual and popular by researchers because it is an ordinary, rapid, and low-cost for the survey of AFM_1_ (1-5). 

The target of our research is to survey the attendance and risk assessment of exposure of AFM_1_ through the consumption of cheese variety in Hamadan province of Iran.

## Experimental

*Sample collection*

Seventy cheeses variety included cream cheese (n = 30) and Iranian white cheese (n = 40) were arbitrarily purchased from popular markets in different markets in Hamadan province, Iran, during from October 2017 to August 2018. Eventually, all of the samples were carried to the lab inside and kept in refrigerator at 4 ^o^C. All cheese samples were analyzed for AFM_1 _before the expiration date of the samples.

*Methods*

The quantitative measurement of AFM_1_ in samples was distinguished by competitive ELISA using AFM_1_ test kit (RIDASCREEN® AFM_1_ Art. No: R1121, R-Biopharm, Darmstadt, Germany). Preparation of the cheeses samples and AFM_1_ measurement were performed according to the method described by kit manufacturer (15). The mean lower limit of detection (LOD) for AFM_1_ in cheese was 5 ng/kg. 

*Risk assessment for exposure to AFM*_1_ *through cheese*

In this study, EDI and HI of AFM_1_ was determined to show the severity and probability of liver cancer risk through cheese consumption (12, 13). EDI was calculated through the following equation:

EDI (ng/kg bw/day) = mean of AFM_1_ in cheese (ng/kg) × average daily consumption of cheese (kg/day)/body weight (kg) 

For calculation of EDI, in the samples in which AFM_1_ concentration was lower than the LOD of ELISA kit (5 ng/kg), it was considered 2.5 ng/kg. Based on the information in the statistical center of Iran, the per capita consumption of cheese has been approximately 13 kg for adults and 8 kg for children in 2018 (16). Average body weight of an adult male, an adult female, and a preschool child was considered as 70, 60, and 20 kg, respectively. 

HI (expressed as ng/kg bw) is calculated as following

HI= EDI (ng/kg bw/day) /0.2 (ng/kg bw/day) 

*Statistical Analysis*

The concentrations of AFM_1_ in milk samples were analysed by SPSS Statistics 16.0 for Windows. One-side t-test was applied to compare the mean concentration of AFM_1_ samples with the maximum acceptable amount of the ISIRI and European Union (250 ng/kg) regulation. Differences between values were considered significant at *P* ≤ 0.05.

## Results

The occurrence and the levels of AFM_1_ in Iranian white cheese samples is summarized in Table 1. 

AFM_1_ was detected above acceptable level in 97.5% (39/40) of the analysed samples, ranging from 5 to 287 ng/kg. Levels of the AFM_1_ in 7 (10%) cheese samples exceeded the ISIRI and European union *i.e*. 250 ng/kg. On the other hand, considering the US FDA (17). limits for AFM_1_ in milk (500 ng/l), none of the samples had levels above the maximum tolerance limit.

EDI of AFM_1_ through both cheese for a preschool child, an adult female and an adult male was 0.138, 0.076, 0.065 ng/kg bw/day, respectively (Table 2). In our study, HI values for a preschool child, an adult female, and an adult male were 0.690, 0.378 and 0.324, respectively. 

## Discussion

AFM_1_ is related with the casein during cheese production making cheese the potent source of AFs among dairy products (18). Cheese is the only production which is sensitive to the development of fungus and mycotoxins groups among the milk products (19). Therefore, the acceptable extent range by regulatory authorities is five to nine folds greater than those ranges for milk (20). The previous studies revealed that some factors such as the action of cheese types, the kind of unit processes and the amount of omitted water pending processing have impacts on the increase of AFM_1 _in cheese sample (21). Also, the several studies by researchers confirmed that cheese samples made from different animal’s milk are effective on amount of AFM_1_. They reported that the level of AFM_1_ from cows’ milk is higher than those of sheep and goats, and this may be because of the differences in their digestive apparatuses and mechanism of aflatoxin B_1_ (AFB_1_) assimilation in animals, and for the different patterns of feeding (19, 22 and 23).

In the current research we show that a high occurrence of AFM_1_ is in various types of cheese including cream and Iranian white cheese from Iran. As referred in previous studies, the occurrence of AFM_1_ in milk and milk derivative contributes to the effects of feeding livestock with materials including aflatoxin B_1_ (6). In a prior survey, Cano-Sancho *et al.* (24) reported the absence of AFM_1_ at detectable level in cheese samples although Altun *et al*. (25) detected AFM_1_ in 100% of cheese samples. Furthermore, there are several research have shown the occurrence of AFM_1_ in some types of cheese such as Tulum, Urfa, Lighvan, Parmesan cheese, Talesh, Halloumi and etc., in Iran, Turkey, Italy, Brazil, Lebanon, and some countries (13, 25-35). These results confirmed that about nutritional importance traditional cheeses between humans and also attention of authorities to this subject. Table 3 shows data regarding AFM_1_ from previous studies in different countries that measure by ELISA and HPLC methods (13, 20, 25-35).

The other obtained results were reported in Pakistan by Iqbal *et al.*, that were done with HPLC technique, from 119 and 150 samples of white cheese and cream cheese, 93 (78%) and 89 (59%) of samples were contaminated with AFM_1_, respectively (36). Also, 14 (15%) samples of white cheese and 10 (11%) cream cheese samples had higher AFM_1_ content than the limit allowed in European Union *i.e* 250 (ng/kg), but our results were less than this result. Also, the previous survey by Elkak *et al.* from Lebanon by ELISA method reported that 75 (67.56%) samples of 111 samples of cheese were detected with AFM_1_ and in 13 (17.33%) samples, concentration of AFM_1_ was higher than the EU regulations (250 ng/kg) (37). This result is approximately to the same as our research results. In other studies, conducted in Iran, the authors also identified Iranian white cheese samples that were contaminated with AFM_1_. According to a study done by Tavakoli *et al.*, from 50 Iranian white cheese samples, collected in Tehran, 60% (30/50) were positive for AFM_1_ at levels of 40.9 to 374 ng/kg detected by ELISA method. Also, 3 (6%) samples were above the permissible level according to ISIRI (4). However, this result is in contrast to our finding that showed 95.71% (67/70) were occurrence of AFM_1_. The other conducted results, that were revealed in Turkey by Bakırdere *et al.*, were observed with ELISA technique, 36 (53.8%) from 67 white cheese and 8 (38%) from 21 cream cheese samples were contaminated with AFM_1 _(38). But, our results reported that approximately all samples of (39/40) white and (28/30) cream cheese are contaminated with AFM_1_. An earlier study by Mohajeri *et al.* from Iran reported that 29 (64.4%) of 45 Iranian white cheese samples were contaminated with AFM_1_ but this result is less than that of the current study (32).

The EDI value in current study was higher than previous reports in Iran and French (13, 39). Shahbazi *et al.* reported EDI value depended on sampling season and AFM_1_ measurement method. The EDI value for AFM_1_ measured by ELISA was 0.04 and 0.03 ng/kg bw/day during winter and summer seasons, respectively (13). However, EDI was 0.05 ng/kg bw/day in winter and 0.04 ng/kg bw/day in summer for this mycotoxin if it was analysed by HPLC method. Leblanc *et al.* reported EDI of 0.02 ng/kg bw/day for AFM_1_ through cheese consumption by French adults (15 years and over) and children (3–14 years) (39).

Because HI level was less than one, it can be concluded that the potential risk for liver cancer in Iranian consumers due to the consumption of Iranian white cheese and cream cheese isn’t a concern. HI value obtained in our study was more than findings reported by Shahbazi *et al.* (13). In the mentioned study, HI values for the cheese samples collected in summer and analysed by ELISE and HPLC were 0.17 and 0.25, respectively while in cheese samples collected in winter, HI was 0.21 by both analysis methods.

**Table 1:** The occurrence and concentration (ng/kg) of AFM_1 _in cheese samples collected from Hamadan province, Iran

**Sample type**	**N**	**Positive (%)**	**Mean**	**Standard deviation**	**< 5**	**5-0.250**	**>250**	**Range**
Iranian white cheese	40	39 (97.50)	115.16	79.22	1 (2.5%)	35 (87.5%)	4 (10%)	< 5-287.09
Cream cheese	30	28 (93.30)	141.20	77.06	2 (6.7%)	25 (83.3%)	3 (10%)	< 5-287.18
Total	70	67 (95.71)	126.32	78.81	3 (4.3%)	60 (85.7%)	7 (10%)	< 5-287.18

**Table 2:** The estimated daily intake (EDI) and hazard index (HI) for AFM_1 _intake through cheese consumption

**Sample type**	**EDI (ng/kg** **bw/day)**			**HI**		
	**Preschool children**	**Female**	**Male**	**Preschool children**	**Female**	**Male**
Iranian white cheese	0.126	0.069	0.059	0.630	0.345	0.296
Cream cheese	0.155	0.085	0.073	0.773	0.423	0.363
Total	0.138	0.076	0.065	0.690	0.378	0.324

**Table 3:** Occurrence and levels of aflatoxin M_1_ (ng/kg) in various cheeses published in previous studies

**Location**	**Cheese type**	**No. of samples**	**No. positive samples (%)**	**Detection Method**	**Mean (ng/kg)**	**Range (ng/kg)**	**Exceeded regulation, n (%)**	**Reference**
Qatar	Cheese	46	39 (84.8)	ELISA	197.74	1.21-217.15	0^a^	Hassan *et al* (20)
Iran	Traditional cheese	360	194 (53.8)	ELISA	139.4	50.5- 308.7	22 (10.5)^a^	Shahbazi *et al* (13)
Turkey	Cheese	130	130 (100)	ELISA	260.26	10-800	22 (17)	Altun *et al* (25)
Iran	Cheese	100	52 (52)	ELISA	133.2	50.2-424.4	8 (8)^a^	Sharifzadeh *et al* (26)
Iran	Cheese	40	25 (65.5)	ELISA	158.4	52.5-272	4 (10)^a^	Bahrami *et al *(27)
Serbia	cheese	54	29 (53.70)	UHPLC-MS/MS	324.07	80-2230	7 (13%)	Škrbić* et al* (28)
Turkey	White pickled cheese	50	10 (20)	ELISA	103.2	40.41-130.89	0^a^	Temamogullari and Kanici (29)
Iran	Cheese	80	69 (86.3)	ELISA	133.2	14.3-572.1	11 (13.8)^a^	Rahimi (30)
Brazil	Cheese	10	3 (30)	HPLC	160	91-300	ND	Jager *et al* (31)
Iran	Lighvan cheese	37	10 (27)	ELISA	90.8	70.5-203	1 (2.7)^b^	Mohajeri *et al* (32)
Italy	Cheese	17	7 (41.2)	HPLC	ND	<3-18	0	Santini *et al* (33)
Iran	Cheese	40	16 (40)	ELISA	133.2	31.9-505.7	7 (17.5)^a^	Nilchian and Rahimi (34)
Turkey	Urfa cheese	127	36 (28.3)	ELISA	253.7	70.61-770.97	13 (10.2)^a^	Kav *et al* (35)

ND: Not Determined; ELISA: Enzyme-Linked Immunosorbent Assay; HPLC: High-Performance Liquid Chromatography.

^a^The European Community limit for aflatoxin M_1_ is 250 ng/kg for cheese

^b^According to Iranian Standard (200 ng/kg).

## Conclusion 

Our finding demonstrated that the incidence of AFM_1 _in cheese samples was high and almost 95.71% samples contained AFM_1_. The results regarding risk assessment indicated HI level was less than one, therefore it can be concluded that potential risk for liver cancer in Iranian consumers due to the consumption of Iranian white cheese and cream cheese isn’t a concern. However, it is essential to set difficult legislations on AFB_1_ contamination in the animal feed. 
